# AN INTEGRATED TRANSITIONAL CARE MODEL FOR OLDER ADULTS WITH MULTIMORBIDITY AND DEPRESSIVE SYMPTOMS

**DOI:** 10.1093/geroni/igz038.3181

**Published:** 2019-11-08

**Authors:** Maureen Markle-Reid, Carrie McAiney, Rebecca Ganann, Kathryn Fisher, Amy Bartholomew, McElhaney Janet, Gauthier Alain, Amiram Gafni

**Affiliations:** 1 McMaster University, Hamilton, Ontario, Canada; 2 University of Waterloo, Waterloo, Ontario, Canada; 3 Health Sciences North, Research Institute, Medical Sciences Division, Northern Ontario School of Medicine, Sudbury, Ontario, Canada; 4 Laurentian University, Sudbury, Ontario, Canada

## Abstract

This pragmatic randomized controlled trial examined the implementation, effectiveness and costs of a nurse-led transitional care intervention to improve hospital-to-home transitions for 127 older adults (≥ 65 years) with depressive symptoms and multimorbidity in three Ontario communities. Participants were randomly allocated to receive the intervention plus usual care (n=63) or usual care alone (n=64). The intervention included an average of 5 in-home visits and 6 phone calls from a Registered Nurse (RN) over a 6-month period. The RN provided system navigation, patient education, medication review, and management of depressive symptoms and chronic conditions. Implementation outcomes included engagement rate, intervention dose, and feasibility of intervention implementation. Effectiveness outcomes included quality of life, depressive symptoms, anxiety, social support, and health and social service use and costs. Participants were an average of 76 years and had an average of 8 chronic conditions. Findings suggest that the intervention was feasible and acceptable to participants and providers. Intention-to-treat analyses using ANCOVA models showed no statistically significant group differences for the outcomes. However, the upper 95% confidence interval for the mean group difference showed greater clinically significant improvements in physical functioning in the intervention group. Quantile regression showed that the intervention may result in greater improvements in physical functioning for individuals with low to average physical functioning values compared to the control group. The intervention may also result in higher levels of perceived social support for individuals with a range of social support values. No statistically significant group differences were observed for service use or costs.

